# AtNHX5 and AtNHX6 Control Cellular K^+^ and pH Homeostasis in *Arabidopsis*: Three Conserved Acidic Residues Are Essential for K^+^ Transport

**DOI:** 10.1371/journal.pone.0144716

**Published:** 2015-12-09

**Authors:** Liguang Wang, Xuexia Wu, Yafen Liu, Quan-Sheng Qiu

**Affiliations:** MOE Key Laboratory of Cell Activities and Stress Adaptations, School of Life Sciences, Lanzhou University, Lanzhou, Gansu, China, 73000; University of Cambridge, UNITED KINGDOM

## Abstract

AtNHX5 and AtNHX6, the endosomal Na^+^,K^+^/H^+^ antiporters in *Arabidopsis*, play an important role in plant growth and development. However, their function in K^+^ and pH homeostasis remains unclear. In this report, we characterized the function of AtNHX5 and AtNHX6 in K^+^ and H^+^ homeostasis in *Arabidopsis*. Using a yeast expression system, we found that AtNHX5 and AtNHX6 recovered tolerance to high K^+^ or salt. We further found that AtNHX5 and AtNHX6 functioned at high K^+^ at acidic pH while AtCHXs at low K^+^ under alkaline conditions. In addition, we showed that the *nhx5 nhx6* double mutant contained less K^+^ and was sensitive to low K^+^ treatment. Overexpression of *AtNHX5* or *AtNHX6* gene in *nhx5 nhx6* recovered root growth to the wild-type level. Three conserved acidic residues, D164, E188, and D193 in AtNHX5 and D165, E189, and D194 in AtNHX6, were essential for K^+^ homeostasis and plant growth. *nhx5 nhx6* had a reduced vacuolar and cellular pH as measured with the fluorescent pH indicator BCECF or semimicroelectrode. We further show that AtNHX5 and AtNHX6 are localized to Golgi and TGN. Taken together, AtNHX5 and AtNHX6 play an important role in K^+^ and pH homeostasis in *Arabidopsis*. Three conserved acidic residues are essential for K^+^ transport.

## Introduction

It is increasingly evident that plant Na^+^,K^+^/H^+^ antiporters (NHX antiporters) are critical for cellular ion homeostasis and pH regulation, and play significant roles in various cellular processes, including Na^+^ and K^+^ movement, pH homeostasis, vesicle trafficking and fusion, regulation of cell cycle and cell proliferation, salt tolerance, and growth and development [1–7].

Na^+^,K^+^/H^+^ antiporters are found in all kinds of life including bacteria, yeast, plants and animals [1, 3, 4, 8, 9]. Na^+^,K^+^/H^+^ antiporters form a large gene family, and presently there are more than 200 genes that have been annotated as Na^+^,K^+^/H^+^ antiporters [9]. The Na^+^,K^+^/H^+^ antiporter is categorized into the monovalent cation proton antiporter (CPA) gene family [9–11]. The *Arabidopsis* genome contains approximately 44 genes that are predicted to encode Na^+^,K^+^/H^+^ antiporters: 8 *AtNHXs*, 28 *AtCHXs* and 6 *AtKEAs*. The *AtNHXs* are members of the CPA1 gene family while the *AtCHXs* and the *AtKEAs* belong to the CPA2 family [1, 3, 4, 10, 12].

Based on their subcellular localizations and anticipated functions, the AtNHX family are divided into three distinct classes: vacuolar (AtNHX1-AtNHX4), endosomal (AtNHX5 and AtNHX6), and plasma membrane (AtNHX7/SOS1and AtNHX8) [1, 3, 4, 9]. The plasma membrane and vacuolar NHXs have been characterized extensively. Studies show that AtNHX7/SOS1 is critical for cellular Na^+^, K^+^ and pH homeostasis, and *sos1* mutants are sensitive to salt stress [13–20]. The vacuolar NHXs are involved in the regulation of cellular ion and pH homeostasis, and play important roles in salt tolerance, K^+^ homeostasis, and plant growth and development [1–3, 8, 21].

The function of the endosomal NHXs are beginning to be explored. A recent report presents that AtNHX5 and AtNHX6 are localized to Golgi and TGN, and may function in endosomal sorting, cellular stress responses, and growth and development in plants [22]. AtNHX5 has been shown to be involved in salt tolerance [23–25]. However, the role of AtNHX5 and AtNHX6 in K^+^ and pH homeostasis remains to be studied.

In this report, we characterized the function of AtNHX5 and AtNHX6 in K^+^ and pH homeostasis in *Arabidopsis*. We found that AtNHX5 and AtNHX6 are crucial to cellular pH and K^+^ homeostasis. We identified three conserved acidic amino acids that are essential for K^+^ homeostasis and plant growth. Our results demonstrate that AtNHX5 and AtNHX6 play an important role in K^+^ and pH homeostasis in *Arabidopsis*.

## Results

### AtNHX5 and AtNHX6 mediate K^+^ and Na^+^ transport in yeast

**Fig 1:**
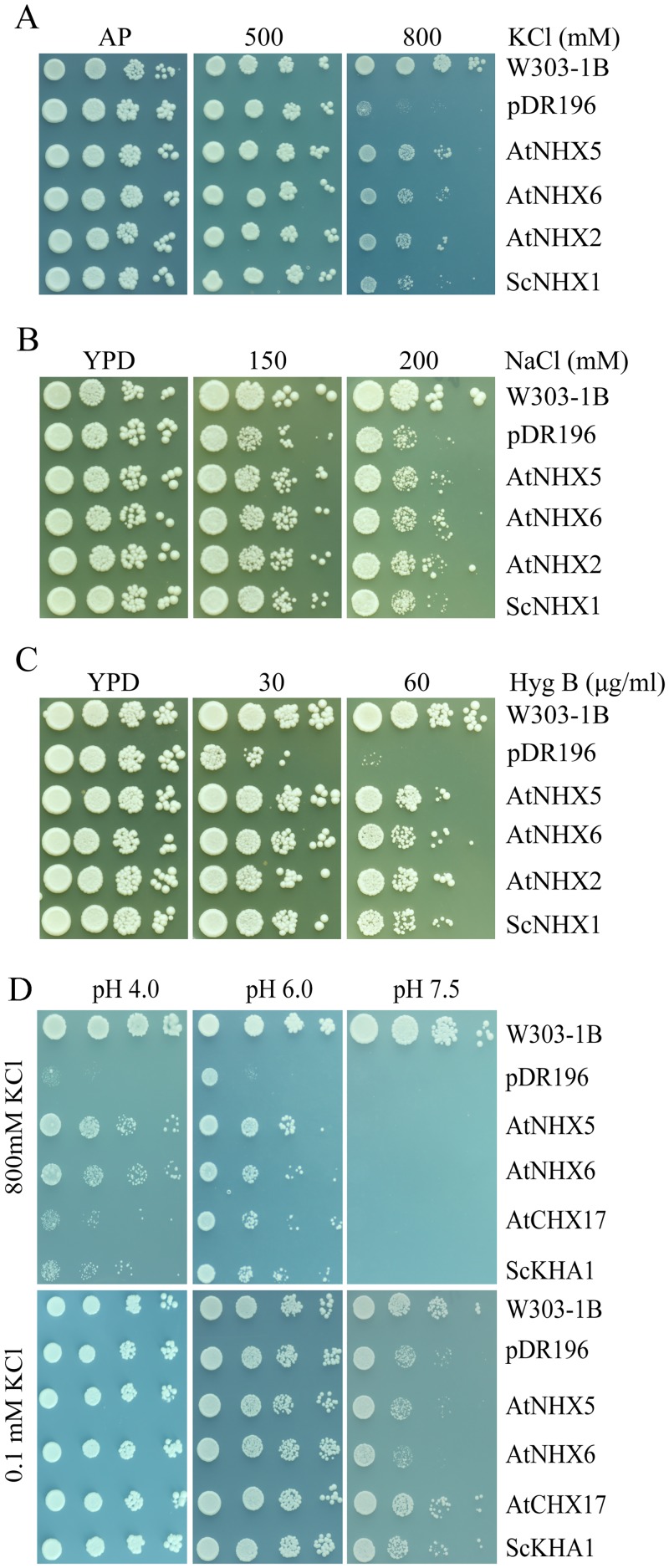
AtNHX5 and AtNHX6 facilitate K^+^ and Na^+^ transport in yeast. The overnight grown yeast cells were normalized in water to A_600_ of 0.12. Aliquos (4 μL) of 10-fold serial dilutions were spotted on AP plates supplemented with KCl (A) or YPD plates with NaCl (B) and Hyg B (C). (D) Aliquos (4 μL) of 10-fold serial dilutions were spotted on AP plates supplemented with 800 mM or 0.1mM KCl at pH 4.0, 6.0 or 7.5. The strains were grown at 30°C for 3 days.

We first tested the ion transport activity of AtNHX5 and AtNHX6 using a yeast expression system. The coding sequences of *AtNHX5 and AtNHX6* were cloned in the yeast expression vector pDR196 and introduced into a *Saccharomyces cerevisiae* strain AXT3. AXT3 lacks the plasma membrane Na^+^-ATPases ScENA1-4, plasma membrane Na^+^,K^+^/H^+^ antiporter ScNHA1, and vacuolar Na^+^,K^+^/H^+^ antiporter ScNHX1. Therefore, it is sensitive to salt and to high K^+^. The yeast was grown on Arg phosphate (AP) or YPD medium containing high levels of KCl or NaCl (Fig 1), respectively. AXT3 mutants failed to grow in the medium containing 800 mM KCl or 200 mM NaCl while the *nhx1*-positive strain W303-1B grew robustly (Fig 1A and 1B). AtNHX5 and AtNHX6 recovered tolerance to high K^+^ or salt, similar to the AXT3 strains expressing ScNHX1 or AtNHX2 (Fig 1A and 1B). In addition, AtNHX5 and AtNHX6 did not confer Li^+^ tolerance (S1 Fig). AXT3 mutants were sensitive to hygromicin B (60 μg/ml), and ScNHX1 and AtNHX2 improved tolerance to hygromicin B (Fig 1C). Expression of AtNHX5 and AtNHX6 conferred resistance to the drug hygromicin B (Fig 1C), implying their roles in endosomal compartments. These results suggest that the endosomal NHX antiporters AtNHX5 and AtNHX6 facilitate both K^+^ and Na^+^ homeostasis and function in endosomal trafficking, which is similar to the vacuolar NHX antiporter AtNHX2. Moreover, AtNHX5 and AtNHX6 showed similar recovery capacities in salt, high K^+^ or hygromicin B treatment, suggesting these two endosomal NHX antiporters share similar catalytic mode for ion transport.

### AtNHX5 and AtNHX6 confer yeast growth at acidic pH

AtNHX5 and AtNHX6 recovered AXT3 mutant growth at 800 mM KCl at pH 6.0 (Fig 1D). However, AtNHX5 and AtNHX6 completely lost their functions at pH 7.5 at 800 mM KCl (Fig 1D). Interestingly, AtNHX5 and AtNHX6 were still active when pH was dropped to 4.0 under 800 mM KCl; however, the recovery capacity of ScKHA1 and AtCHX17 was significantly reduced under the same conditions (Fig 1D). AtNHX5 and AtNHX6 were further tested in the yeast mutant strain AXT4K, generated by deleting *kha1* in the AXT3 background. AXT4K mutants failed to grow at low K^+^ at pH 7.5, while the *kha1*-positive strain W303-1B grew vigorously (Fig 1D). However, expression of AtNHX5 and AtNHX6 failed to confer AXT4K growth at low K^+^ at pH 7.5, while ScKHA1 and AtCHX17 improved yeast growth (Fig 1D). These results suggest that AtNHXs and AtCHXs may have different modes of action in mediating K^+^ homeostasis. AtNHXs function at high K^+^ at acidic pH while AtCHXs at low K^+^ under alkaline conditions.

### AtNHX5 and AtNHX6 are essential to K^+^ homeostasis in *Arabidopsis*

We next generated the *nhx5 nhx6* double mutant to characterize the function of AtNHX5 and AtNHX6 in K^+^ homeostasis in *Arabidopsis*. We obtained one T-DNA line for the *AtNHX5* gene (*nhx5-1*) and two separate T-DNA lines for the *AtNHX6* gene (*nhx6-1* and *nhx6-2*) (S2 Fig). The double knockout lines were produced by crossing *nhx5-1* with *nhx6-1* and *nhx6-2*, respectively, to obtain two independent double knockout lines, *nhx5-1 nhx6-1* and *nhx5-1 nhx6-2*. The absence of the *AtNHX5* and *AtNHX6* transcripts in these double knockout lines was confirmed by RT-PCR (S2 Fig). These two double knockout lines had identical growth phenotypes (S3 Fig). The *nhx5-1 nhx6-1* double mutant line was used in the following experiments.

Similar to Bassil et al. (2011), we found that the *nhx5 nhx6* double mutant showed profound defects in growth and development (S4 Fig). The *nhx5 nhx6* double mutant had smaller rosettes and shorter seedlings, was flowering and bolting late, and produced less seeds (S4 Fig). These results confirmed the notion that AtNHX5 and AtNHX6 play an important role in plant growth and development [26].

To verify the function of the AtNHX5 and AtNHX6 genes, we performed genetic complementation test. GFP was fused with the C-terminus of the AtNHX5 and AtNHX6 genes and the resulting constructs were introduced into the *nhx5 nhx6* double mutants. Transformation of *nhx5 nhx6* with either ANHX5 or AtNHX6 rescued the *nhx5 nhx6* phenotype, and the growth and development of the transgenic seedlings resembled the wild-type seedlings (S4G and S4H Fig). However, overexpression of either AtNHX5 or AtNHX6 in wild-type plants did not enhance plant growth (S4H Fig).

**Fig 2:**
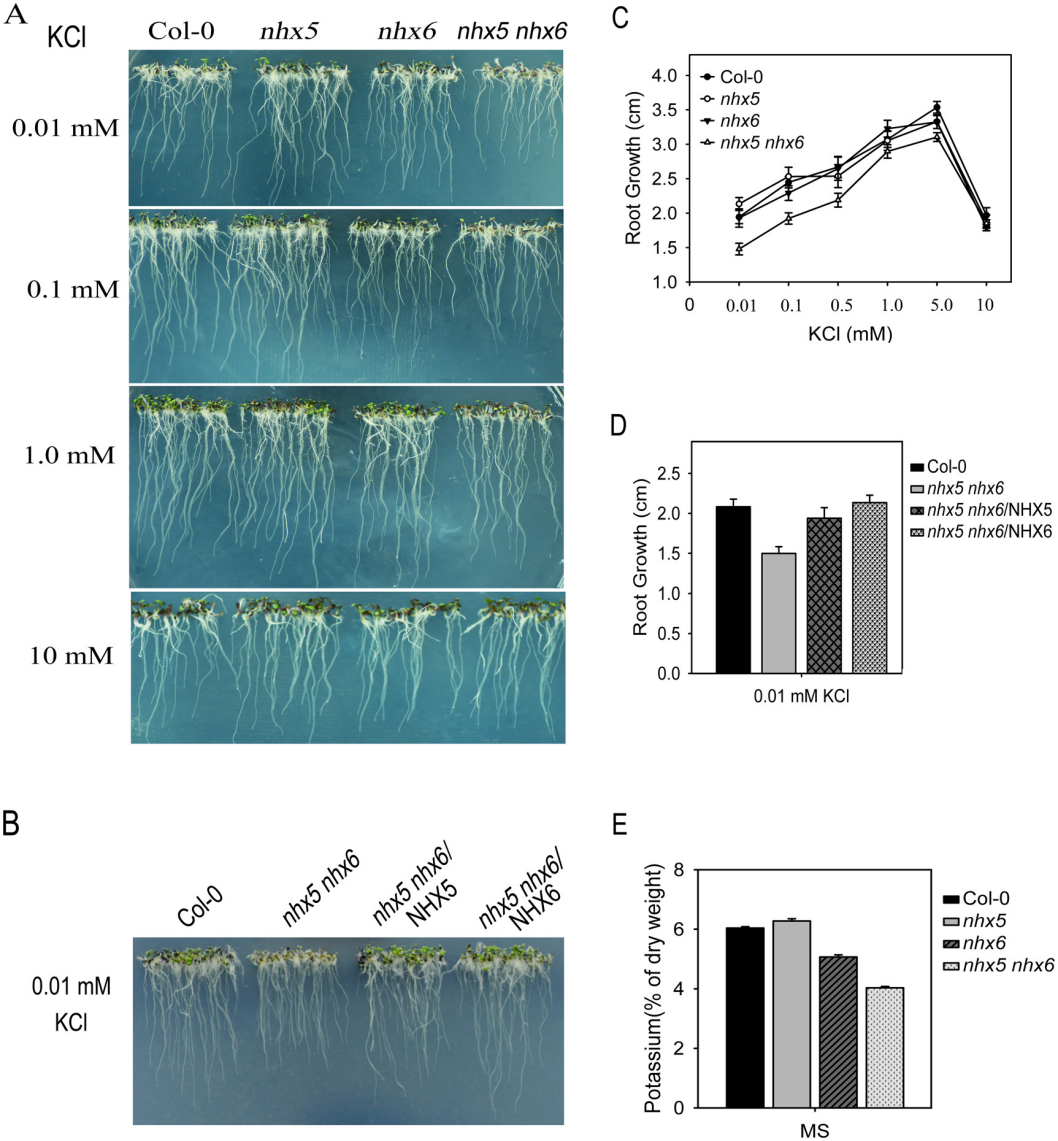
*nhx5 nhx6* is sensitive to low K^+^ treatment. (A) Seedling growth under low K^+^ treatment. (B) Expression of *AtNHX5* and *AtNHX6* recovered *nhx5 nhx6* growth under low K^+^ treatment. (C) Root growth was measured 7 days after the seedlings were grown on the media containing various levels of KCl. Three independent experiments were performed, and about 10 seeds were counted for each experiment. Data are means SD. (D) Root growth was measured 10 days after the seedlings were grown on the media containing 0.01mM KCl. Three independent experiments were performed, and about 10 seeds were counted for each experiment. Data are means SD. (E) K^+^ content in seedlings. Three independent experiments were performed, and about 10 seeds were counted for each experiment. Data are means SD.

To test their function in K^+^ homeostasis in plants, we examined the growth of *nhx5 nhx6* seedlings on media containing various levels of K^+^. The low K^+^ media were made by adding different amount of KCl (from 0.01 to 10 mM) to the potassium-free modified MS medium [27]. The modified MS medium contains 1/20 strength major salts and 1×minor salts. For both the wild-type and mutant seedlings, root growth was slower when the potassium concentration was lower than 1 mM (Fig 2A and 2C). Root growth was peaked at 5 mM KCl, but was reduced dramatically when KCl concentrations were increased to 10 mM for all the seedlings (Fig 2A and 2C). Root growth of the *nhx5* and *nhx6* single mutants was not significantly inhibited compared with the wild-type seedlings (Fig 2A and 2C). However, root growth of *nhx5 nhx6* was inhibited drastically at low potassium levels tested (Fig 2A and 2C). Moreover, overexpression of either the *AtNHX5* or *AtNHX6* genes in *nhx5 nhx6* recovered root growth to the wild-type levels (Fig 2B and 2D). These results suggest that AtNHX5 and AtNHX6 are critical to K^+^ homeostasis in *Arabidopsis*.

In addition, we determined the K^+^ concentrations of the seedlings by the atomic absorption spectrophotometer. The K^+^ levels in *nhx5 nhx6* were dramatically reduced when grown in MS media (Fig 2E). Interestingly, the *nhx6* single mutant had a reduced K^+^ while *nhx5* was not affected significantly (Fig 2E).

### Conserved acidic residues in AtNHX5 and AtNHX6 are critical for K^+^ transport in *Arabidopsis*

Studies from bacteria, yeast and mammals show that acidic residues in transmembrane domains of Na^+^/H^+^ antiporters are critical for exchange activity [28–30]. Sequence alignment reveals that there are four conserved acidic residues in the transmembrane domains of Na^+^/H^+^ antiporters [31]. Mutation of these conserved acidic residues in yeast ScNhx1p blocked protein trafficking in yeast. These studies suggest that the conserved acidic amino acids in Na^+^/H^+^ antiporters are critical for exchange activity as well as cellular functions [31].

**Fig 3:**
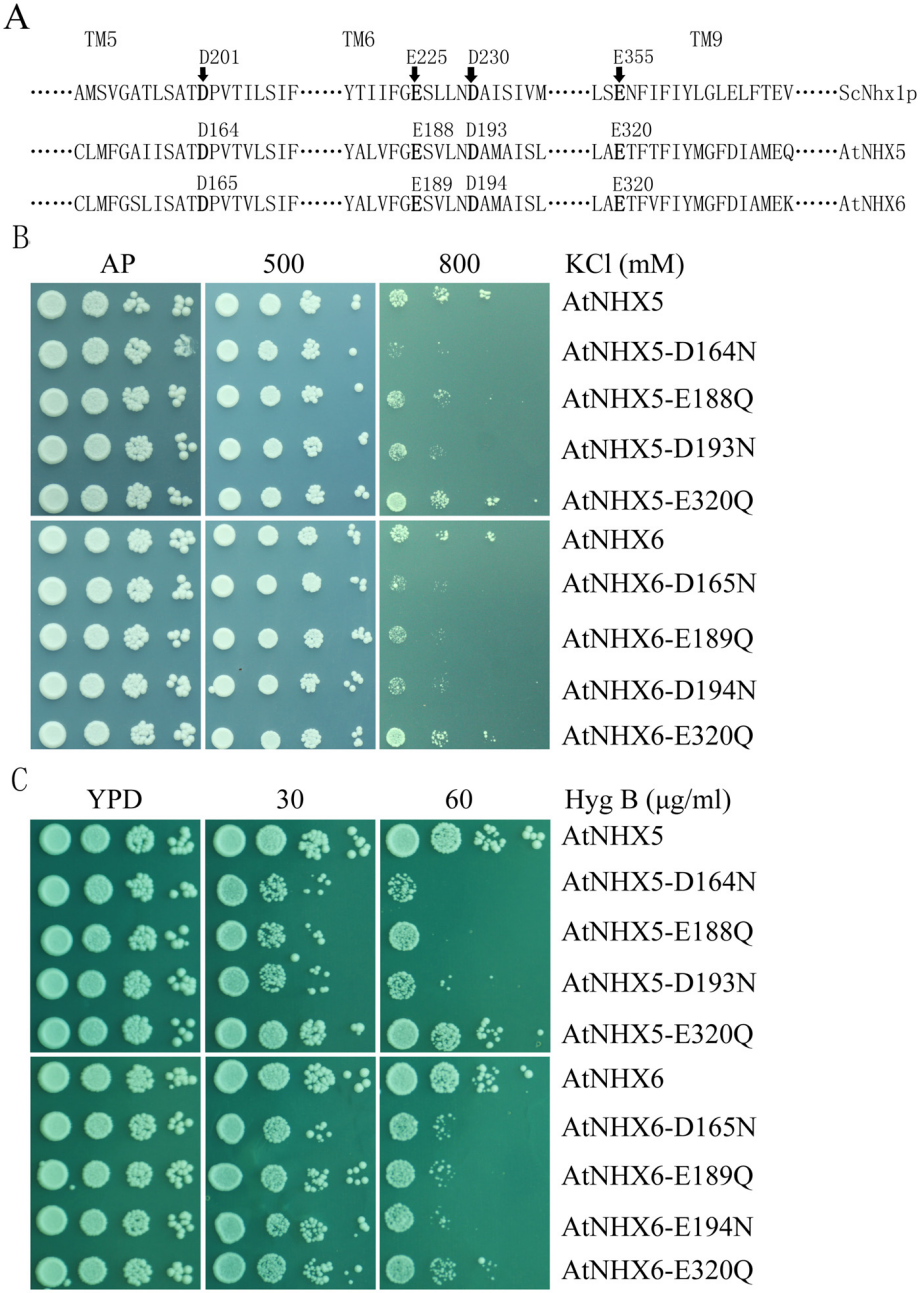
Three conserved acidic residues in AtNHX5 and AtNHX6 are critical for K^+^ transport in yeast. (A) Sequence alignment of AtNHX5 and AtNHX6 with yeast (*S*. *cerevisiae*) ScNhx1p identified four conserved acidic amino acids in the transmembrane domains of AtNHX5 and AtNHX6. The alignment was made by using the complete amino acid sequences, but only the predicted transmembrane domains 5, 6, and 9 are shown. (B) and (C) Yeast growth test for the point mutants of AtNHX5 and AtNHX6. The yeast strains were grown overnight in AP medium. Yeast cells were normalized in water to A_600_ of 0.12. Aliquos (4 μL) of 10-fold serial dilutions were spotted on AP plates supplemented with KCl (B) or on YPD plates with Hyg B (C). The strains were grown at 30°C for 3 days.

We are interested in identifying whether the plant NHXs have the same conserved acidic residues that are essential for their exchange activity and cellular functions. Interestingly, AtNHX5 and AtNHX6 contain the conserved acidic residues in transmembrane domains when aligned with the ScNhx1p sequence (Fig 3A). The four acidic residues D164, E188, D193 and E320 of AtNHX5 align with the D201, E225, D230 and E355 of ScNhx1p, respectively; similarly, the D165, E189, D194 and E320 of AtNHX6 line up with the D201, E225, D230 and E355 of ScNhx1p, respectively (Fig 3A).

To test their function, we substituted these four acidic amino acids with uncharged polar residues. In AtNHX5, D164 was mutated to N, E188 to Q, D193 to N and E320 to Q; in AtNHX6, D165 to N, E189 to Q, D194 to N and E320 to Q. The mutant genes of *AtNHX5* and *AtNHX6* were cloned in the yeast vector pDR196 and introduced into the strain AXT3. The yeast was grown on Arg phosphate (AP) or YPD medium containing high levels of KCl or hygromicin B (Fig 3B and 3C), respectively. Surprisingly, the D164 N, E188 Q and D193 N mutants of AtNHX5 lost their capability in recovering yeast growth in both high K^+^ and hygromicin B (Fig 3B and 3C). Similarly, the D165N, E189Q and D194 N mutants of AtNHX6 failed to recover yeast growth in high K^+^ and hygromicin B (Fig 3B and 3C). These results suggest that the conserved acidic residues are essential for ion transport activity and cellular functions of the plant NHXs. Furthermore, the plant NHXs may share the same catalytic mechanism for ion transport as their bacterial, yeast and mammalian counterparts. However, the E320Q mutants of both AtNHX5 and AtNHX6 remained their activities in conferring yeast growth under both high K^+^ and hygromicin B (Fig 3B and 3C). This is similar to the observation that the ScNhx1p mutant E355Q is still active in protein trafficking in yeast (Bowers et al., 2000), suggesting that this conserved glutamic acid may not be involved in exchange activity and cellular functions.

**Fig 4:**
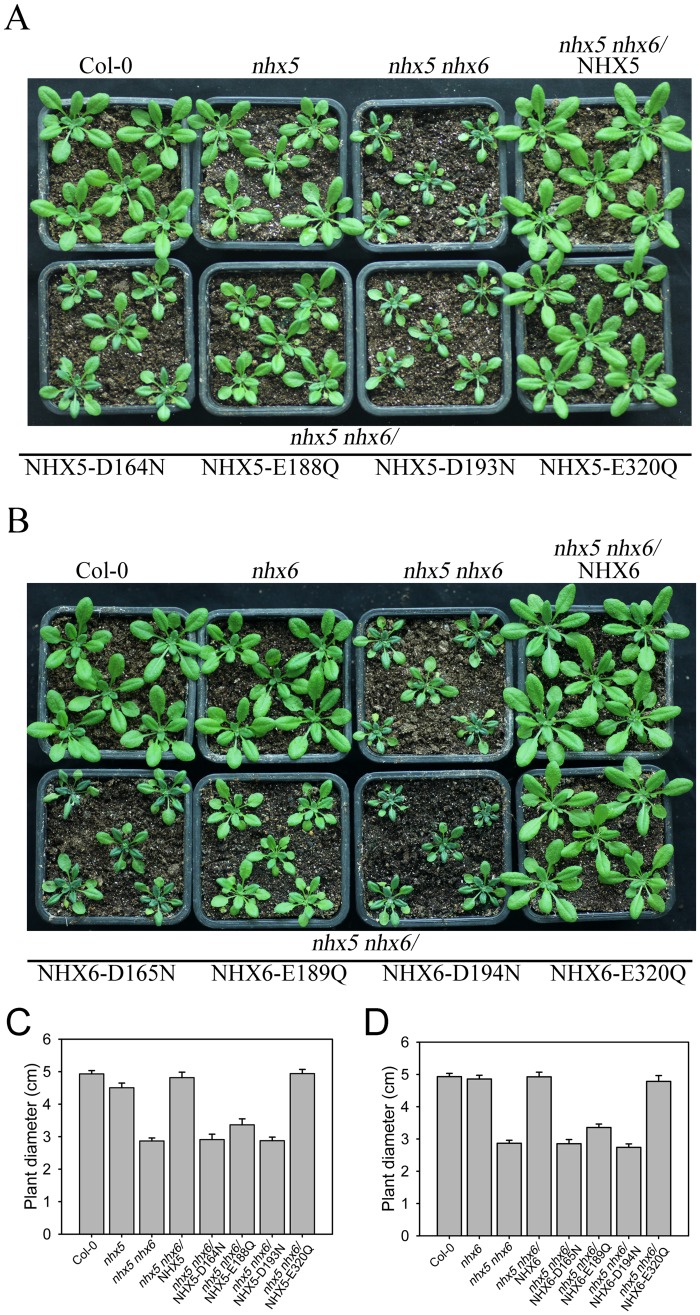
Three conserved acidic residues in AtNHX5 and AtNHX6 are essential for the growth and development in *Arabidopsis*. (A) and (B) Growth phenotype of the transgenic *Arabidopsis* seedlings bearing point mutants of AtNHX5 and AtNHX6. The point mutated genes of the four conserved acidic residues of AtNHX5 and AtNHX6 were introduced into the *nhx5 nhx6* background. Photos were taken for the T3 seedlings grown in soil for 26 d. (C) and (D) The rosette size of the transgenic Arabidopsis seedlings bearing point mutants of AtNHX5 and AtNHX6. The rosette sizes were measured when the seedlings were grown in soil for 26 d.

To further test the function of the conserved acidic residues in growth and development in *Arabidopsis*, we expressed *AtNHX5* and *AtNHX6* genes mutated in these four conserved residues in *nhx5 nhx6* double mutant seedlings. The mutations were made by replacing the acidic residues with uncharged polar residues (as described in the yeast test). The genes were cloned into pUBC-GFP plasmids, driven by Ubiquitin-10 (Ub10) promoter. GFP was fused with the C-termini of the genes. Intriguingly, consistent with the yeast test, the D164 N, E188 Q and D193 N mutants of AtNHX5 failed to complement the growth of the *nhx5 nhx6* seedlings, although a partial recovering was observed in D164 N and E188 Q mutants of AtNHX5 (Fig 4A and 4C). Likewise, the D165N, E189Q and D194 N mutants of AtNHX6 did not complement the growth of the *nhx5 nhx6* seedlings (Fig 4B and 4D). A partial recovering was observed in E189Q mutant of AtNHX6 (Fig 4B and 4D). Similar to the yeast test, the E320Q mutants of both AtNHX5 and AtNHX6 fully recovered the growth of the *nhx5 nhx6* seedlings (Fig 4A–4D). These results suggest that the conserved acidic amino acids play essential roles in growth and development in *Arabidopsis*.

### AtNHX5 and AtNHX6 regulate cellular pH homeostasis

**Fig 5:**
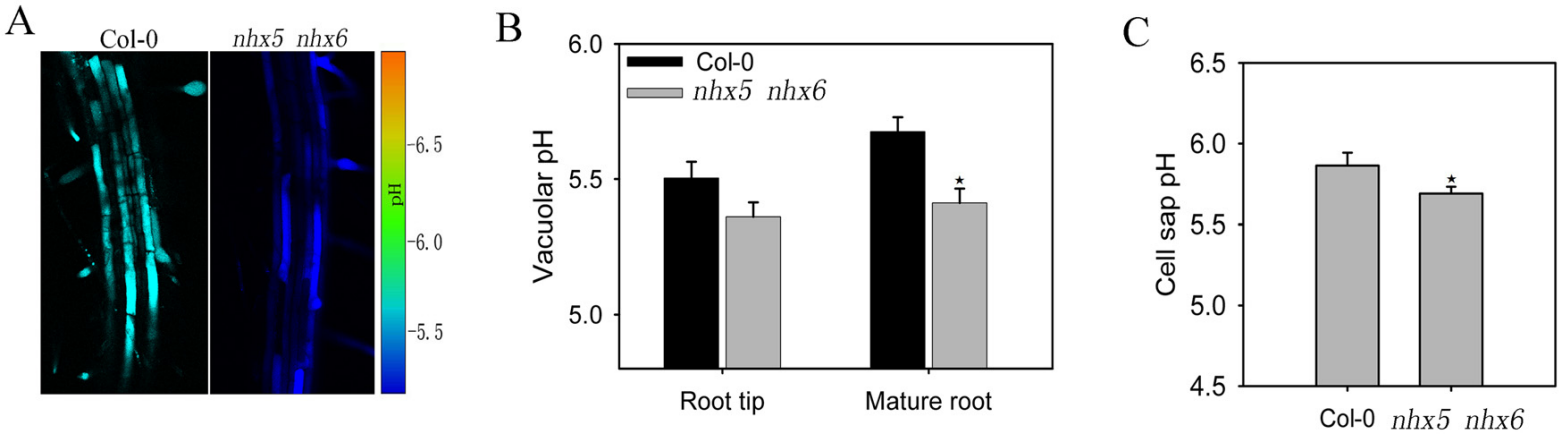
*nhx5 nhx6* has a reduced cellular pH. (A) Ratio images of the epidermal cells of the mature root zone in Col-0 and *nhx5 nhx6*. (B) Vacuolar pH was reduced in epidermal cells of the mature root. The vacuolar pH was measured using the fluorescein-based ratiometric pH indicator BCECF. Error bars represent SD of 30 measurements from 15 seedlings. Asterisks indicate significant difference (P≤ 0.05; *t* test). (C) The cell sap pH of *nhx5 nhx*6 was reduced. The cell sap pH was measured from 4-week-old plants. Error bars show SD of three independent experiments. Asterisks indicate significant difference (P≤ 0.05; *t* test).

To test the function of AtNHX5 and AtNHX6 in pH regulation, we measured vacuolar pH using an imaging-based approach (Fig 5) [22, 32]. The fluorescein-based ratiometric pH indicator BCECF (2′,7′-bis-(2-carboxyethyl)-5-(and-6)-carboxyfluorescein) was loaded into vacuoles of intact roots using its membrane-permeant acetoxymethyl (AM) ester. pH values were calculated from fluorescence ratios of confocal images using an in situ calibration curve (S5 Fig). Fig 5A shows the representative ratio images of the BCECF-stained vacuolar fluorescence in mature roots of the wild-type and *nhx5 nhx6* seedlings. For the root tip cells, no significant difference was observed in vacuolar pH between the wild-type (pH 5.50) and *nhx5 nhx6* (pH 5.36) seedlings (Fig 5B). However, the vacuolar pH was significantly reduced in mature roots of the *nhx5 nhx6* seedlings (pH 5.41) compared with the wild-type (pH 5.68) seedlings (Fig 5B).

To verify the function of AtNHX5 and AtNHX6 in facilitating pH homeostasis, we measured the cellular pH with a different approach. We extracted the cell sap from rosette leaves and measured the pH using a semimicroelectrode [32–34]. Consistent with the BCECF measurement, the cell sap pH of the *nhx5 nhx6* seedlings was significantly reduced to 5.69, while the wild-type seedlings had a pH of 5.86 (Fig 5C).

### AtNHX5 and AtNHX6 are Localized to Golgi and TGN in *Arabidopsis*

**Fig 6:**
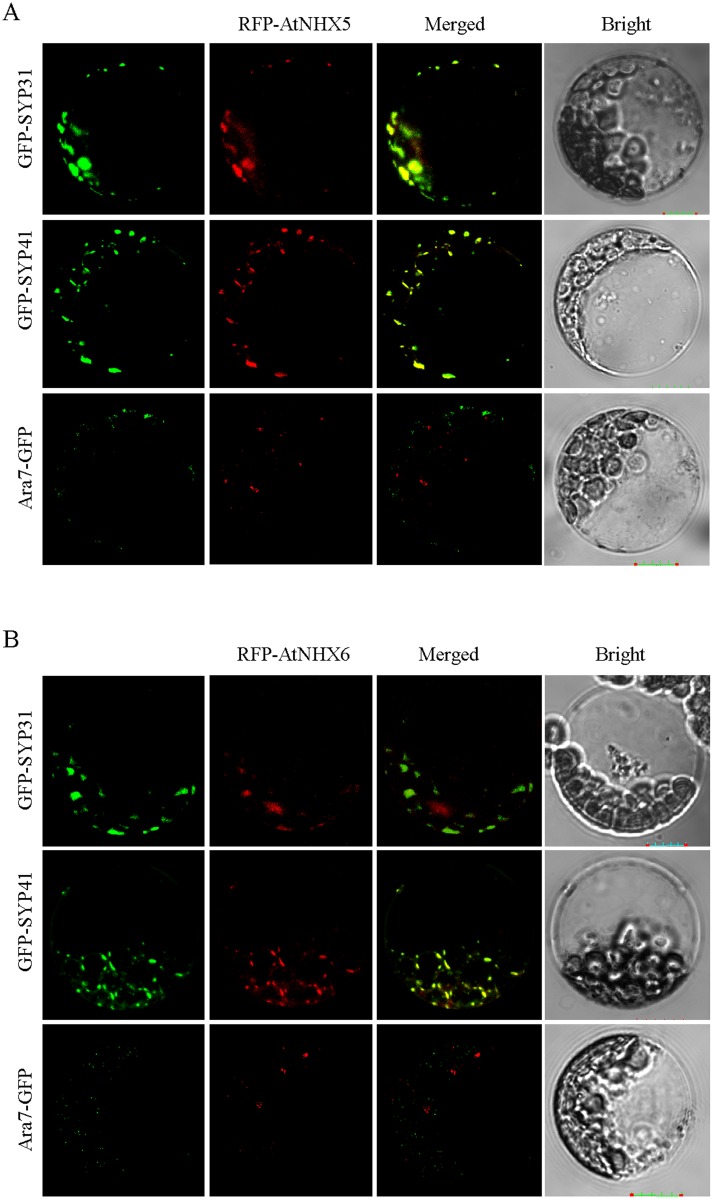
Subcellular localization of AtNHX5 and AtNHX6 in the *Arabidopsis* protoplasts. RFP-AtNHX5 and RFP-AtNHX6 were co-localized with the Golgi marker GFP-SYP31and TGN marker GFP-SYP41. The Golgi marker GFP-SYP31, TGN marker GFP-SYP41 or prevacuolar compartment marker Ara7-GFP was co-transformed with the RFP-AtNHX5 or RFP-AtNHX6, respectively. (A) Subcellular localization of AtNHX5. (B) Subcellular localization of AtNHX6. Scale bar = 10 μm.

The subcellular localization of AtNHX5 and AtNHX6 was first visualized by transient expression in *Arabidopsis* protoplasts. RFP genes were fused to the N-terminal ends of *AtNHX5* and *AtNHX6*, driven by the 35S promoter. The *RFP-AtNHX5* or *RFP-AtNHX6* plasmids were transiently co-expressed in *Arabidopsis* leaf protoplasts with Golgi marker GFP-SYP31, TGN marker GFP-SYP41 or PVC marker Ara7-GFP. RFP-AtNHX5 fluorescence appeared on punctate structures in the cytosol (Fig 6A). RFP-AtNHX5 fluorescent signals were co-localized extensively with AtSYP31-GFP and GFP-SYP41 but not Ara7-GFP, suggesting that AtNHX5 is localized to Golgi and TGN (Fig 6A). Similarly, RFP-AtNHX6 fluorescence also appeared on punctate structures and its signals were co-localized with AtSYP31-GFP and GFP-SYP41 but not Ara7-GFP, suggesting that AtNHX6 is also localized to Golgi and TGN (Fig 6B).

**Fig 7:**
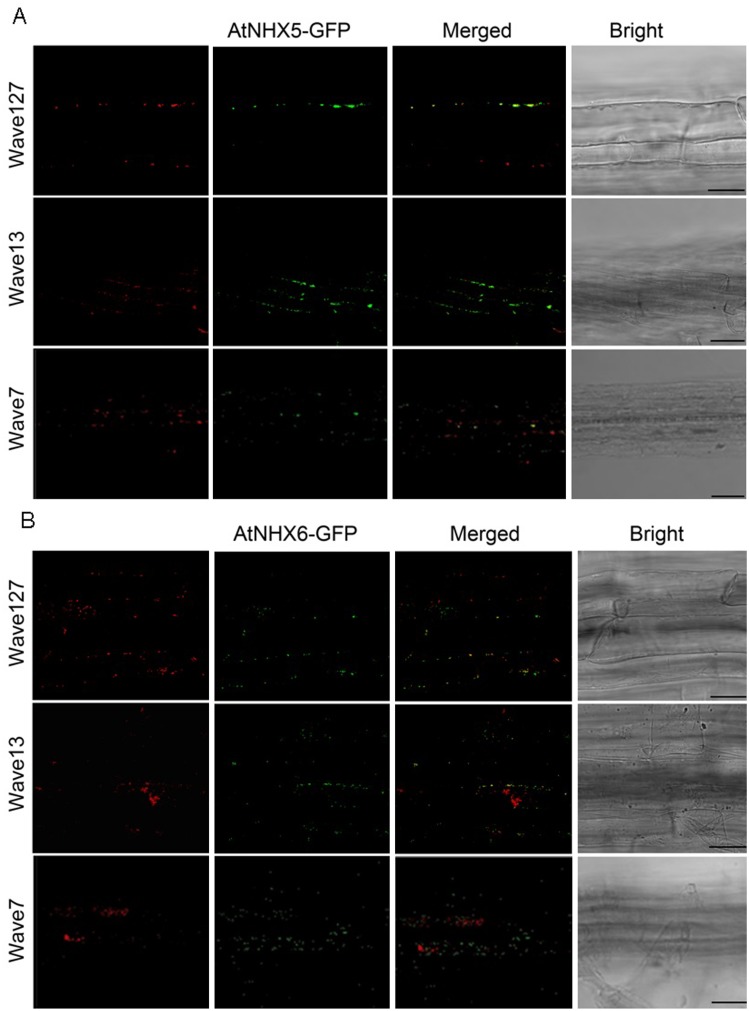
Subcellular localization of AtNHX5 and AtNHX6 in stably transformed *Arabidopsis*. Double reporter lines were generated by co-expressing the Golgi marker Wave127 (mCherry- MEMB12), TGN marker Wave13 (mCherry-VTI12), or PVC marker Wave7 (mCherry-Rha1) with either AtNHX5-GFP or AtNHX6-GFP. (A) Subcellular localization of AtNHX5. (B) Subcellular localization of AtNHX6. Scale bar = 25 μm.

The subcellular localization of AtNHX5 and AtNHX6 was verified by stably transformed *Arabidopsis* seedlings co-expressed various organelle markers. The double reporter lines were generated by crossing the AtNHX5-GFP and AtNHX6-GFP seedlings with the Golgi marker Wave127 (mCherry-MEMB12), TGN marker Wave13 (mCherry-VTI12), or PVC marker Wave7 (mCherry-Rha1). In consistent with the transient expression assays, AtNHX5-GFP and AtNHX6-GFP fluorescent signals were visualized at the punctate structures within the cells (Fig 7A and 7B). AtNHX5-GFP and AtNHX6-GFP fluorescent signals were co-localized with mCherry-MEMB12, mCherry-VTI12 but not mCherry-Rha1 (Fig 7A and 7B). These studies from both the transiently expressed protoplasts and stably transformed seedlings suggest that AtNHX5 and AtNHX6 are localized to Golgi and TGN.

## Discussion

### The endosomal K^+^,Na^+^/H^+^ antiporters AtNHX5 and AtNHX6 are crucial for cellular K^+^ and pH homeostasis in *Arabidopsis*

In this report, we characterized the function of AtNHX5 and AtNHX6 in K^+^ and H^+^ homeostasis in *Arabidopsis*. Using a yeast expression system, we found that AtNHX5 and AtNHX6 recovered tolerance to high K^+^ or salt (Fig 1). We showed that the *nhx5 nhx6* double mutant contained less K^+^ and was sensitive to low K^+^ treatment (Fig 2). Overexpression of *AtNHX5* or *AtNHX6* gene in *nhx5 nhx6* recovered root growth to the wild-type level (Fig 2B and 2D). In addition, *nhx5 nhx6* had a reduced vacuolar and cellular pH as measured with the fluorescent pH indicator BCECF or semimicroelectrode (Fig 5). We further show that AtNHX5 and AtNHX6 are localized to Golgi and TGN. These results indicate that AtNHX5 and AtNHX6 function in facilitating K^+^ transport in cells and play an important role in K^+^ and pH homeostasis in *Arabidopsis*.

Sequence analysis indicates that AtNHXs share high similarity among their transmembrane domains [23]. Since the transmembrane domains participate in the transport of cations and H^+^ across the membrane, the conservative in transmembrane domains among the AtNHX family may suggest that the AtNHX members share similar mechanisms for ion transduction. Indeed, all types of NHXs, including the endosomal NHXs [this study and 22], vacuolar NHXs [35–40], and plasma membrane NHXs [15, 16, 19], have been demonstrated to participate in K^+^ and Na^+^ transport in *Arabidopsis*. These results suggest that AtNHXs share a common mode of action and are involved in K^+^ and Na^+^ transport in *Arabidopsis*.

*Arabidopsis* contains three different types of Na^+^,K^+^/H^+^ antiporters, including AtNHXs, AtKEAs and AtCHXs, but their action mode for ion transport is still less defined. Phylogenetic analysis shows that AtNHXs, AtKEAs and AtCHXs form three distinct clusters with their E. coli or yeast orthologs [41], suggesting that these three different types of Na^+^,K^+^/H^+^ antiporters may function distinctly from each other in plants. In this study, using yeast growth assay, we found that AtNHX5 and AtNHX6 function at high K^+^ at acidic pH while AtCHXs at low K^+^ under alkaline conditions (Fig 1D). In addition, we have shown in a previous study that AtKEAs have strict pH requirements and function at high K^+^ at pH 5.8, suggesting that AtKEAs may function differently from both AtNHXs and AtCHXs [41]. Furthermore, we found that similar to the plasma membrane and vacuolar NHXs, AtNHX5 and AtNHX6 recovered yeast tolerance to salt stress (Fig 1B), suggesting that AtNHXs may function in salt stress. Nevertheless, neither AtCHXs nor AtKEAs have been found to improve yeast growth in salt stress [4, 41]. These results suggest that AtNHXs, AtKEAs and AtCHXs may have different modes of action in mediating K^+^ or Na^+^ transport.

### Three conserved acidic residues in AtNHX5 and AtNHX6 are important for K^+^ transport and seedling growth in *Arabidopsis*

Bowers et al. (2000) identified four conserved acidic residues in transmembrane domains of NHE proteins from species including yeast, plants, human beings, insects and rats. Mutation of three of these residues blocked protein transport out of the PVC, suggesting that these conserved amino acids are crucial for vacuolar trafficking in yeast. These conserved residues may be vital for ion exchange activity since mutation of E262 in human NHE1 (E262 is equivalent to E225 of yeast ScNhx1p) abolished ion exchange activity [28]. A homology modeling study shows that two of the residues (Asn262 and Asp267) are localized within TM5. TM5 is located close to TM4 and TM11, which form an assembly structure and involve in conformation change at the cation-binding site following pH activation. Thus, localization of these acidic residues in the proximity of the core structure suggests that they may function in binding and translocating cations in the process of ion exchange.

In this study we found that AtNHX5 and AtNHX6 contain four conserved acidic amino acids in transmembrane domains that align with the ScNhx1p and human NHE1 sequences (Fig 3A). We showed that mutation of three of the conserved residues in both AtNHX5 and AtNHX6 failed to recover yeast growth in high K^+^ and hygromicin B (Fig 3B and 3C). We further expressed the mutated genes of these conserved residues in AtNHX5 and AtNHX6 in *nhx5 nhx6*, and demonstrated that the mutants failed to complement the growth of the *nhx5 nhx6* seedlings as well (Fig 4A and 4B). Our results suggest that the conserved acidic residues play critical roles in K^+^ transport and growth and development in *Arabidopsis*. These results also suggest that AtNHX5 and AtNHX6 may share similar core structure and transport mode to their yeast and human counterparts, and these conserved acidic residues may involve in binding and translocating cations in ion exchange.

## Materials and Methods

### Plant materials and growth conditions

*Arabidopsis thaliana* ecotypes Columbia (Col-0), mutants, and transgenic lines were used in this study. In the growth chamber, plants were grown on compost (Pindstrup Substrate, Latvia) and subirrigated with tap water. Greenhouse conditions were as follows: 16-h-light /8-h-dark cycles, light intensity 100 μmol s^-1^ m^-2^ photosynthetically active radiation, temperature 22°C, and relative humidity 50 ± 10%. For plate-grown plants, *Arabidopsis* thaliana seeds were surface sterilized with 20% (v/v) bleach. After cold treatment at 4°C for 3 days in the dark, the seeds were germinated on plates with Murashige and Skoog (MS) medium containing 1.0% agar, pH 5.8. For growth at low potassium, seedlings grew on the modified MS medium containing various concentrations of KCl. The modified MS medium contains 1/20 strength major salts and 1×minor salts. For solidification of MS medium, 1.0% ultra-pure agarose was used [27].

### Generation of the *nhx5 nhx6* double mutant and complementation assays

T-DNA insertion lines were obtained from the SALK collection. Alleles and SALK lines used in this work were Wisc-DsLox345-348M8 (*nhx5-1*), SALK_113129C (*nhx6-1*), and SALK_100042 (*nhx6-2*). Insertion mutant information was obtained from the SIGnAL website (http://signal.salk.edu) and confirmed experimentally. Positions of T-DNA insertion sites are shown in S2A Fig online. Mutant *nhx5-1* has a T-DNA insertion at nucleotide +1504 relative to the start codon. The T-DNA insertion in line *nhx6-1* occurred at nucleotide +3809 relative to the start codon, whereas mutant *nhx6-2* carries the insertion at nucleotide +545. Homozygous mutant lines were identified by PCR screening with allele-specific primers designed to amplify wild-type or mutated loci.

The double mutant lines were generated by crossing *nhx5-1* with *nhx6-1* or *nhx6-2* to obtain *nhx5-1 nhx6-1* and *nhx5-1 nhx6-2*, using *nhx6-1* or *nhx6-2* as pollen donors. The homozygous double mutant lines were identified by PCR screening with allele-specific primers designed to amplify wild-type or mutated loci.

For complementation assays, the CDS of *AtNHX5* and *AtNHX6* (without stop codons) was amplified by PCR from pDR196-NHX5 and pDR196-NHX6, ordered from ABRC, using the following primers: AtNHX5 (5’-ACAAGTTTGTACAAAAAAGCAGGCTTCATGGAGGAAGTGATGATTTCTCCG-3’ and 5’-ACCACTTTGTACAAGAAAGCTGGGTCCTCCCCATCTCCATCTCCATCTC-3’), and AtNHX6 (5’-ACAAGTTTGTACAAAAAAGCAGGCTTCATGTCGTCGGAGCTGCAGATT-3’ and 5’-ACCACTTTGTACAAGAAAGCTGGGTCGCCGCGGTTATTTAGATTTCCTCTT-3’). The PCR products were cloned into the vector pDONR/Zeo (Invitrogen). The entry vectors (pDONR-NHX5 and pDONR-NHX6) were recombined into the expression vector pUBC-GFP using Gateway technology (Invitrogen) to produce pUBC-NHX5-GFP and pUBC-NHX6-GFP. The expression constructs were transformed into the *A*. *tumefaciens* strain GV3101, and the resulting bacterial clones were used to transform *nhx5-1 nhx6-1* for complementation assays by the floral dip procedure [42]. Meanwhile, the expression constructs were introduced into *Arabidopsis* Col-0 plants to generate the overexpression plants. Transgenic plants were screened on MS medium supplemented with 0.0015% Basta. The homozygous lines of T3 progeny were selected for experiments.

### Generation of the point mutants of the conserved acidic residues in AtNHX5 and AtNHX6

For plant transformation, pDONR-NHX5 and pDONR-NHX6 were used as templates to generate the point mutants. The site-directed mutagenesis was performed by Quikchange mutagenesis. For *AtNHX5*, the mutations were GAC to AAT (pDONR-NHX5-D164N), GAA to CAA (pDONR-NHX5-E188Q), GAT to AAT (pDONR-NHX5-D193N), and GAA to CAA (pDONR-NHX5-E320Q). The mutations of *AtNHX6* were GAT to AAT (pDONR-NHX6-D165N), GAA to CAA (pDONR-NHX6-E189Q), GAT to AAT (pDONR-NHX6-D194N), and GAG to CAA (pDONR-NHX6-E320Q). These point mutants of *AtNHX5* and *AtNHX6* were recombined into pUBC-GFP using Gateway technology. The C-termini of these genes was fused with GFP, driven by Ubiquitin-10 (Ub10) promoter. These plasmids were transformed into the GV3101 *A*. *tumefaciens* strain, and the resulting bacterial clones were used to transform the *A*. *thaliana* (ecotype Columbia) by the floral dip procedure [42]. Transgenic plants were screened *in vitro* on MS medium supplemented with 0.0015% Basta. The homozygous lines of T3 progeny were selected for experiments.

For the yeast expression assay, the point mutants of AtNHX5 and AtNHX6 were amplified by PCR using the pDONR vectors mentioned above as templates. These point mutants were amplified using the following primers: AtNHX5 (5’- CCGGAATTCATGGAGGAAGTGATG -3’ and 5’- CCGCTCGAGCTACTCCCCATCT -3’), AtNHX6 (5’- CCGGAATTCATGTCGTCGGAGCT -3’ and 5’- CCGCTCGAGTTAGCCGCGGTTATTTAG -3’). The PCR products were digested with EcoRI and XhoI and cloned into the yeast vector pDR196.

### Quantitative real-time RT-PCR (RT-qPCR) analysis

Col-0 seedlings growing on MS plates were collected at 7-, 14- and 21-day growth. The total RNA was isolated using the RNAiso Plus (TaKaRa). The first-strand cDNA was synthesized from the total RNA (1 μg) using the PrimeScript^®^ RT reagent kit with cDNA Eraser (TaKaRa), and was used as templates for PCR amplification. PCR amplification was performed with the CFX96 system (Bio-Rad) using the SYBR^®^ Premix Ex Taq^™^ (TaKaRa). The *Arabidopsis* Actin7 gene was used as an internal control, and differences in product levels among the tested samples during the linear amplification phase were used to calculate the differential gene expression [43]. The gene-specific primers used are as follows: AtNHX5 (5′-CATGATCTACCAGAGGGTCACG-3′ and 5′-CAGACATGGAGTCATCAAGATCG-3′), AtNHX6 (5′-GGAAGTGGATTCAGGACAAAAC-3′ and 5′-GTTGCTCCATGTTACCCTCATC-3′).

### Determination of K^+^ content

The mutants and wild-type seedlings grew on MS plates for 10 days. The seedlings were then collected, washed briefly for 4 times with deionized water, and dried at 80°C for 36 h. Dried samples were digested with HNO_3_, and the K^+^ concentration was determined by the atomic absorption spectrophotometer [39].

### Vacuolar pH measurement

The pH-sensitive fluorescent dye BCECF-AM was used to measure the vacuolar pH in root cells [26, 32]. 5-day-old seedlings grown on vertical plates were collected and incubated in liquid media containing 1/10 MS medium, 0.5% sucrose, 10 mM MES (pH 5.8),10 μM BCECF-AM and 0.02% pluronic F-127 (Molecalur probes) for 1 h at 22°C in darkness. The seedlings were washed once for 10 min before microscopy. Dye fluorescent images were obtained using a Leica confocal. The fluorophore was excited at 458 and 488 nm, and single emission between 530 and 550 nm was detected for all the images. The root tip cells and mature root cells of fully elongation zone were collected for the images. After background correction, the integrated pixel intensity was measured for both the 458 and the 488 nm excited images. The ratio values were used to calculate the pH based on the calibration curve, which was calculated using the WCIF ImageJ. For in situ pH calibration, the 5-day-old seedlings were incubated for 15 min in pH equilibration buffers containing 50 mM Mes-BTP (pH 5.3–6.4) or 50 mM Hepes-BTP (pH 6.8–7.6) and 50 mM ammonium acetate [44,45].The average ratio values were determined from 20 individual seedlings.

### Cell Sap pH measurement

The cell sap was extracted from rosette leaves of 4-week-old plants. Leaves dissected were squeezed in a 1.5 ml reaction tube with a micropestle for 2 min. Samples were centrifuged at 20,000 x g for 1 min. The supernatant was transferred to a new tube, and the procedure was repeated once. Pool the supernatants of two extractions and immediately measure the pH of the solution using a semimicroelectrode [32–34]

### Yeast strains, media, and growth conditions

*Saccharomyces cerevisiae* strains W303-1B (MATα *leu2-13 112*, *ura3-1*, *trp1-1*, *his3-11 15*, *ade2-1*, *can1-100*), AXT3 (*ena1-4Δ*::*HIS3*, *nha1Δ*::*LEU2*, *nhx1Δ*:: *TRP1*) and AXT4K (*ena1-4Δ*::*HIS3*, *nha1Δ*::*LEU2*, *nhx1Δ*:: *TRP1*, *kha1Δ*::*KanMX6*) were gifts from Dr. Jose M. Pardo [46–48]. All strains used were derivatives of W303-1B. *Saccharomyces cerevisiae* strain *Δnhx1* was derivatives of BJ3505 (MATα *ura3-52 trp1-Δ101 his3-Δ200 lys2-801 gal2 (gal3) can1 prb1-Δ1*.*6R pep4*::*HIS3*). Untransformed strains were grown at 30°C in YPD medium (1% yeast extract, 2% peptone and 2% glucose). Transformation of yeast cells was performed by the lithium acetate method. After transformation, strains were grown on selective Hartwell's complete (SC) medium or APG medium (10mM arginine, 8mM phosphoric acid, 2 mM MgSO_4_, 1 mM KCl, 0.2 mM CaCl_2_, 2% glucose, and trace minerals and vitamins). NaCl, KCl, or hygromycin B was added to the medium. Drop test media contained 20 mM MES, and pH was adjusted to 7.5 with arginine [49] or to acidic pH values with phosphoric acid [50].

### Functional expression of AtNHX5 and AtNHX6 in yeast

The cDNAs of *AtNHX5*, *AtNHX6* and *AtNHX2*, ordered from ABRC, were cloned into the yeast expression vector pDR196 with the promoter PMA1.

To clone *AtCHX17*, gene fragments were amplified by PCR from *Arabidopsis* cDNA using the following primers: AtCHX17 (5′-AAACTGCAGATGGGAACAAACGGTACAAC-3′ and 5′-CGCGTCGACCTAAGGACTCTCAGAATCC-3′). To clone *ScNHX1* and *ScKHA1*, gene fragments were amplified by PCR from the genomic DNA isolated from the *Saccharomyces cerevisiae* strain BJ3505 using the following primers: ScNHX1 (5′-CGCGTCGACATGCTATCCAAGGTATTGC-3′ and 5′-CCGCTCGAGCTAGTGGTTTTGGGAAGAG-3′), ScKHA1 (5′-CGCGTCGACATGGCAAACACTGTAGGAG-3′ and 5′-CCGCTCGAGTTATTCAGACGAAAAATGGTG-3′). The PCR fragments were cloned into the plasmid pDR196. All gene fragments were verified by sequencing.

All plasmids were transformed into the yeast strain AXT3 or AXT4K; the empty vector pDR196 was transformed into the same yeast strains as a control. For stress tolerance tests, yeast cells were normalized in water to A_600_ of 0.12. 4 μL aliquots of each 10-fold serial dilution were spotted onto AP plates supplemented with KCl, or YPD plates supplemented with NaCl as indicated, and incubated at 30°C for 3 days. Resistance to hygromycin B was assayed in YPD medium.

### Localization of AtNHX5 and AtNHX6 in *Arabidopsis* Protoplasts

The transient expression in protoplasts was performed as described [51]. The protoplasts were derived from the leaf mesophyll cells of *Arabidopsis*. RFP gene was fused in frame to the N-termini of *AtNHX5* and *AtNHX6*. The *AtNHX5* and *AtNHX6* were amplified using the following primers: AtNHX5 (5’-TCGCGGATCCATGGAGGAAGTGATGATT-3’ and 5’- CCCGGAATTCCTACTCCCCATCTCCATC-3’), AtNHX6 (5’- ATCGCGGATCCATGTCGTCGGAGCT-3’ and 5’- TCCGGAATTCTTAGCCGCGGTTATTTAGAT-3’). The amplified fragments were cloned into the EcoRI and BamHI sites of the pSAT6-C1-Red fluorescent protein (RFP) vector to yield the final plasmids RFP-AtNHX5 and RFP-AtNHX6. Plasmids carrying genes tagged with a fluorescent marker were transiently co-expressed with TGN marker GFP-SYP41, Golgi marker GFP-SYP31 and PVC marker Ara7-GFP in leaf protoplasts as described [51,52]. *Arabidopsis* seedlings of 4 weeks old were used for protoplast isolation. Fluorescence was visualized by a confocal laser scanning microscope (FV1000, Olympus). The excitation wavelength was 488 nm for GFP and 594 nm for RFP, and emission was 500–530 nm for GFP and 590–630 nm for RFP.

### Localization of AtNHX5 and AtNHX6 in Stably Transformed *Arabidopsis* Seedlings

For the stable transformation assays with *Arabidopsis thaliana*, the pUBC-NHX5-GFP and pUBC-NHX6-GFP were transformed into *Agrobacterium tumefaciens* GV3101. *Arabidopsis thaliana* (ecotype Columbia) wild-type plants were transformed [42]. The transgenic plants were screened by basta spray; the basta positive seedlings were re-confirmed with PCR amplification of the GFP fragment.

To generate the double reporter lines co-expressing Rha1, VTI12 or MEMB12 with pUBC-NHX5-GFP or pUBC-NHX6-GFP, the homozygous parents were crossed using mCherry-Rha1, mCheery-VTI12 or mCherry-MEMB12 as pollen donors. GFP and mCherry fluorescence were visualized under a confocal laser scanning microscope (FV1000, Olympus). The excitation wavelength was 561 nm and emission was 595–620 nm for mCherry. The roots of the transgenic seedlings containing the Rha1, VTI12 or MEMB12 with pUBC-NHX5-GFP or pUBC-NHX6-GFP fusion protein were visualized under the confocal microscope.

## Supporting Information

S1 Fig AtNHX5 and AtNHX6 do not confer Li^+^ tolerance. (TIF)

S2 Fig Molecular genetics analyses of *nhx5* and *nhx6* mutants. (TIF)

S3 Fig The growth phenotype of *nhx5-1 nhx6-2* double mutant grown on soil for 30 d. (TIF)

S4 Fig The *nhx5 nhx6* double mutant is defective in growth and development. (TIF)

S5 Fig pH calibration curve of BCECF-AM dye loaded roots. (TIF)
