# Supplementary figures and images for: AtNHX5 and AtNHX6 Control Cellular K+ and pH Homeostasis in Arabidopsis: Three Conserved Acidic Residues Are Essential for K+ Transport
Source: PLoS One. 2015 Dec 9;10(12):e0144716. doi: 10.1371/journal.pone.0144716 (PMC4674129; doi:10.1371/journal.pone.0144716)

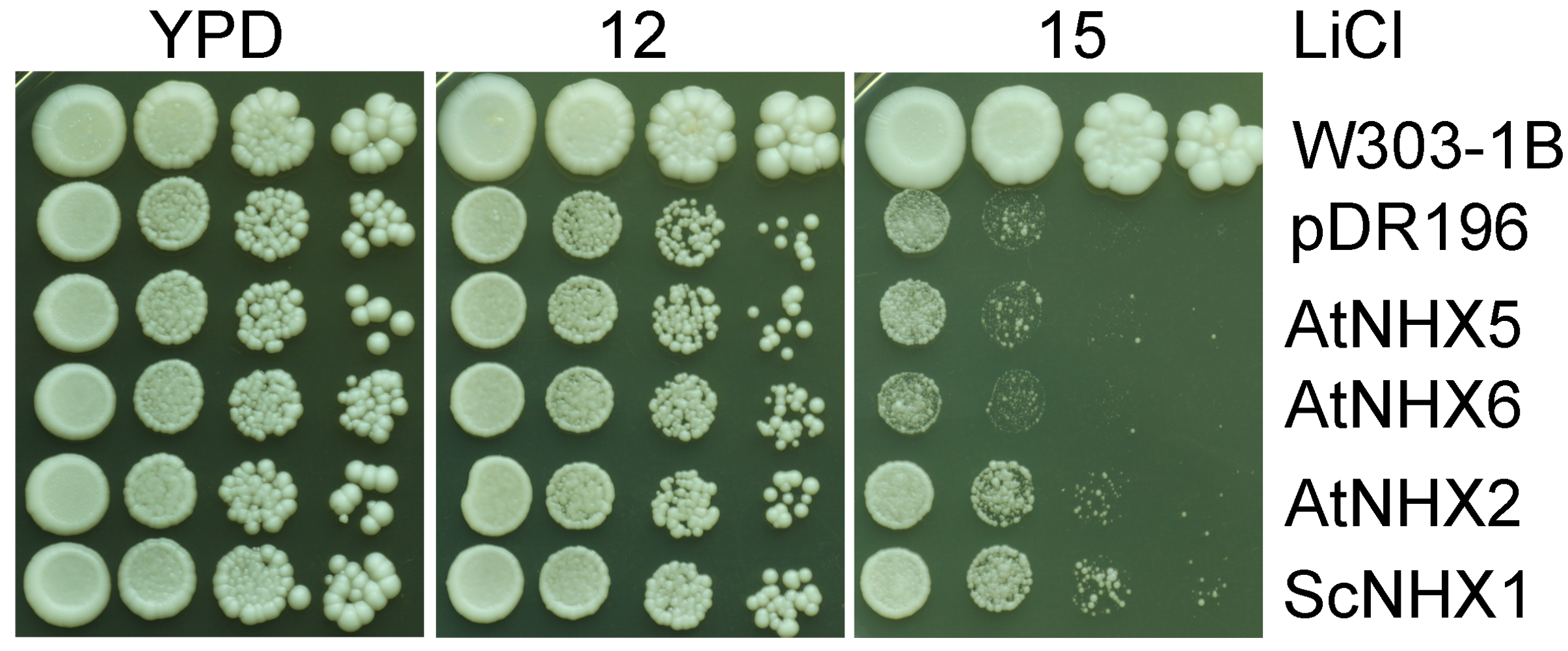

Supplement: S1 Fig — (TIF) [file pone.0144716.s001.tif]

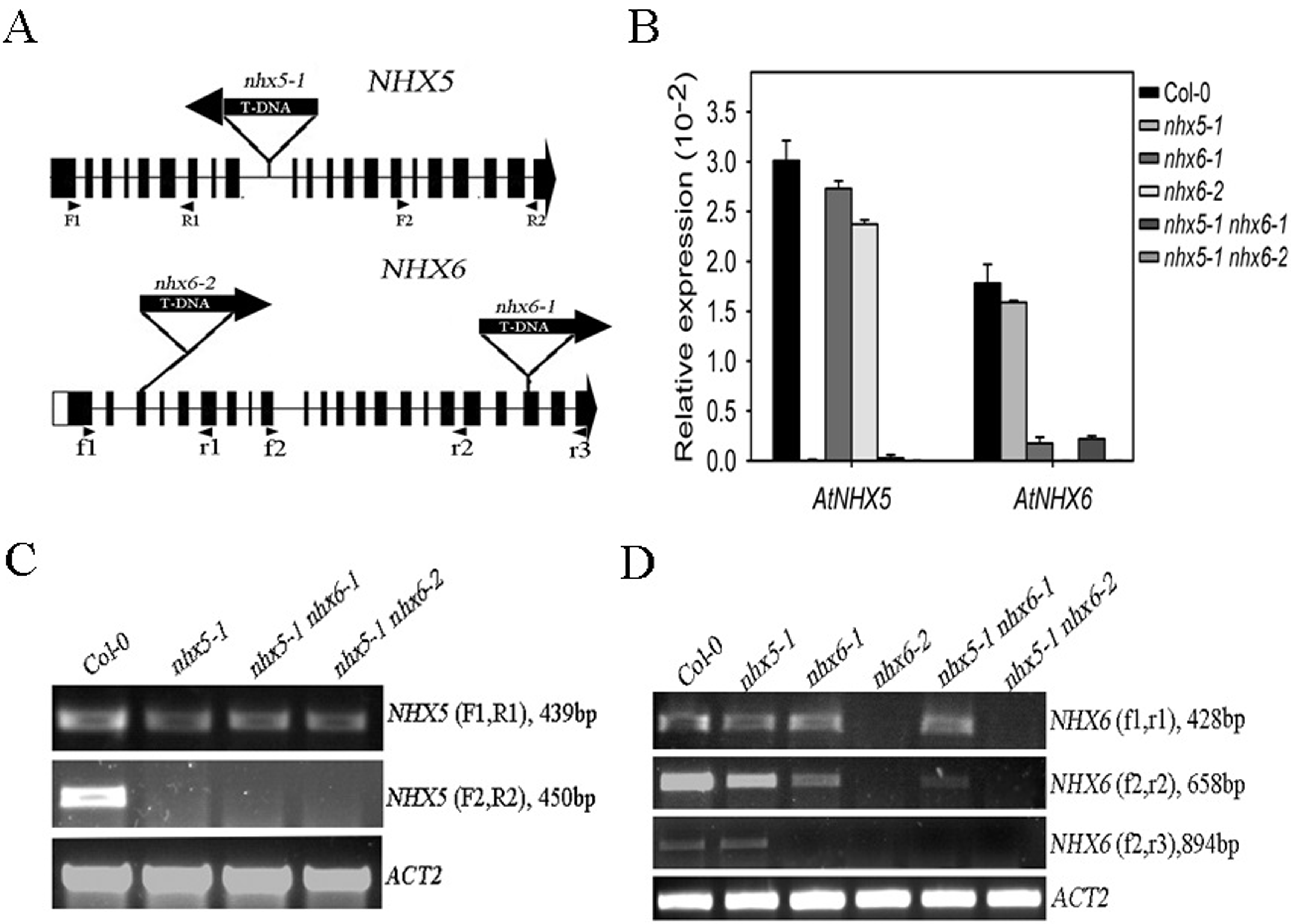

Supplement: S2 Fig — (TIF) [file pone.0144716.s002.tif]

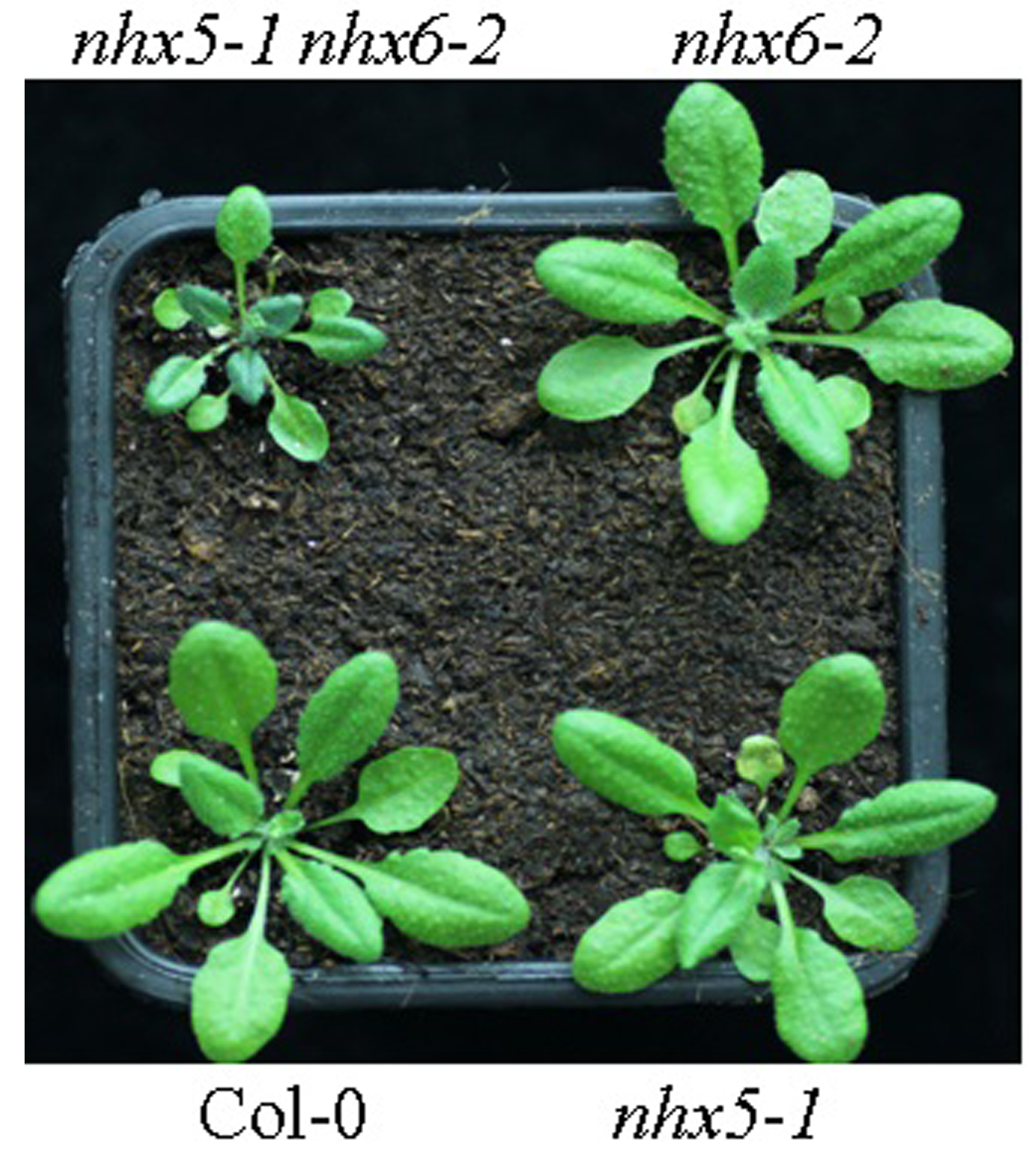

Supplement: S3 Fig — (TIF) [file pone.0144716.s003.tif]

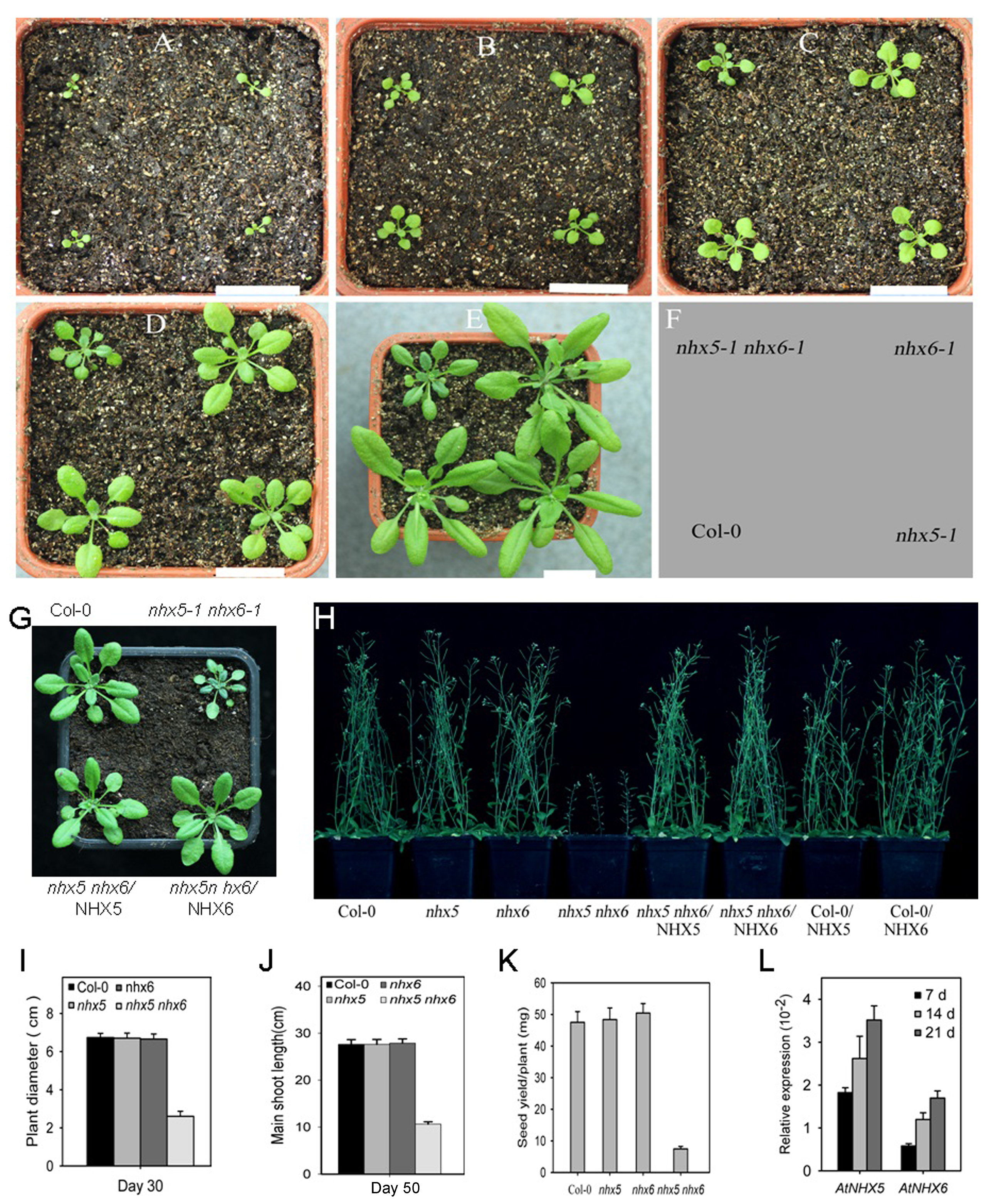

Supplement: S4 Fig — (TIF) [file pone.0144716.s004.tif]

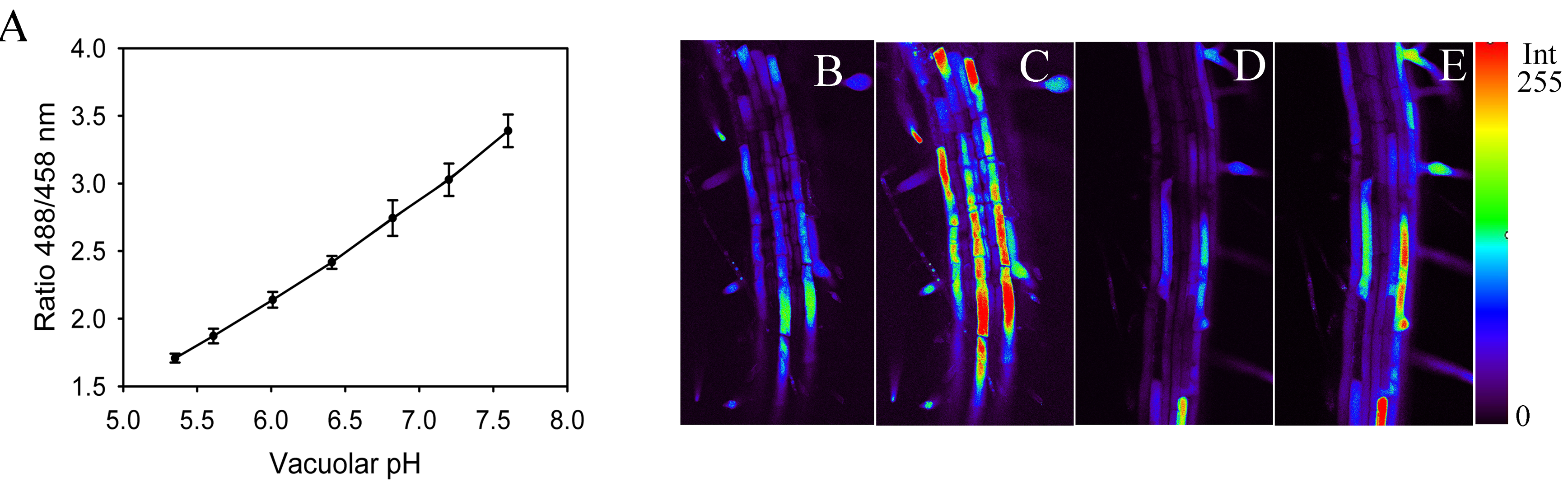

Supplement: S5 Fig — (TIF) [file pone.0144716.s005.tif]
